# Hybrid morphological-convolutional neural networks for computer-aided diagnosis

**DOI:** 10.3389/frai.2023.1253183

**Published:** 2023-09-19

**Authors:** Martha Rebeca Canales-Fiscal, José Gerardo Tamez-Peña

**Affiliations:** ^1^Tecnológico de Monterrey, Escuela de Ingeniería y Ciencias, Monterrey, NL, Mexico; ^2^Tecnológico de Monterrey, Escuela de Medicina y Ciencias de la Salud, Monterrey, NL, Mexico

**Keywords:** deep learning, medical image classification, mathematical morphology, medical image datasets, computer-aided diagnosis

## Abstract

Training deep Convolutional Neural Networks (CNNs) presents challenges in terms of memory requirements and computational resources, often resulting in issues such as model overfitting and lack of generalization. These challenges can only be mitigated by using an excessive number of training images. However, medical image datasets commonly suffer from data scarcity due to the complexities involved in their acquisition, preparation, and curation. To address this issue, we propose a compact and hybrid machine learning architecture based on the Morphological and Convolutional Neural Network (MCNN), followed by a Random Forest classifier. Unlike deep CNN architectures, the MCNN was specifically designed to achieve effective performance with medical image datasets limited to a few hundred samples. It incorporates various morphological operations into a single layer and uses independent neural networks to extract information from each signal channel. The final classification is obtained by utilizing a Random Forest classifier on the outputs of the last neural network layer. We compare the classification performance of our proposed method with three popular deep CNN architectures (ResNet-18, ShuffleNet-V2, and MobileNet-V2) using two training approaches: full training and transfer learning. The evaluation was conducted on two distinct medical image datasets: the ISIC dataset for melanoma classification and the ORIGA dataset for glaucoma classification. Results demonstrate that the MCNN method exhibits reliable performance in melanoma classification, achieving an AUC of 0.94 (95% CI: 0.91 to 0.97), outperforming the popular CNN architectures. For the glaucoma dataset, the MCNN achieved an AUC of 0.65 (95% CI: 0.53 to 0.74), which was similar to the performance of the popular CNN architectures. This study contributes to the understanding of mathematical morphology in shallow neural networks for medical image classification and highlights the potential of hybrid architectures in effectively learning from medical image datasets that are limited by a small number of case samples.

## 1. Introduction

Artificial intelligence (AI) has revolutionized medical image analysis, playing a crucial role in supporting diagnoses. Two prominent approaches have emerged: one involves hand-crafted features coupled with traditional machine learning, while the other leverages convolutional neural networks (CNNs). The latter approach has gained preference due to its ability to automatically learn and extract relevant features, eliminating the need for extensive manual feature engineering (Sarvamangala and Kulkarni, 2022). In the realm of CNNs, numerous architectures have been proposed, many of which boast an extensive number of layers and parameters. ResNet (He et al., 2016), Inception networks (Szegedy et al., 2016, 2017), MobileNet (Sandler et al., 2018), ShuffleNet (Ma et al., 2018), and DenseNet (Huang et al., 2017) are some of the widely adopted architectures in various medical imaging applications (Esteva et al., 2017; Walsh et al., 2018; Lee et al., 2019; Khalifa et al., 2020; Mei et al., 2020; Souid et al., 2021).

In addition to the widely discussed CNN architectures in the literature, there have been proposals to enhance the functionality of CNNs by combining them with traditional machine-learning approaches (Wang et al., 2019; Taherkhani et al., 2020; Deepak and Ameer, 2021). Moreover, alternative feature extraction techniques such as the Gray-Level Co-occurrence Matrix (GLCM), Gabor filters (Jia et al., 2020), local binary patterns (LBP) (Wetzer et al., 2018), and morphological operations (Franchi et al., 2020) have been explored. Interestingly, Mellouli et al. (2019) found that combining convolution and morphology leads to improved recognition performance compared to using these techniques separately. Despite the numerous applications of mathematical morphology in medical imaging (Bhateja et al., 2019; García-Floriano et al., 2019; Zhao et al., 2021), there is a lack of studies that explore its potential within neural networks for medical image classification.

It is suspected that the impact of mathematical morphology would depend on the operations chosen and the specific medical case. Two diseases that could potentially benefit from particular morphological operations are glaucoma and melanoma. In the case of glaucoma, detecting this eye condition involves recognizing specific morphological characteristics within fundus images, i.e., the relationship between optic disc and cup sizes, the optic nerve of the affected eye has an irregular amount of optic nerve cupping (Iqbal et al., 2022). Conversely, typical indicators of melanoma include asymmetry of the lesion, irregular borders, variability in colors, diameter larger than 5mm, and presence of nodular components, all components present in dermoscopic images (Garbe et al., 2022). Both conditions heavily rely on the detection of distinct structural features. Considering that mathematical morphology forms the basis of morphological image processing – often employed to highlight or remove desired geometrical structures – we hypothesized that this capability can potentially aid in identifying the aforementioned features crucial for detecting glaucoma and melanoma.

While different CNN architectures have shown success in various medical image classification tasks, they also come with certain drawbacks. One challenge is the optimization of hyperparameters, which can be a complex task. Additionally, to capture low-level textural information in images, small-sized kernels are preferred, but this choice increases the computational complexity during training. Moreover, CNNs with significant depth and a large number of parameters require substantial memory and computational resources, making training computationally intensive. These factors often contribute to inadequate training, leading to issues such as model overfitting and a lack of generalization. To address the challenges of overfitting and generalization, an extensive number of training images are required, and these extensive sets are lacking in many medical conditions.

The limited availability of large datasets for training CNNs poses significant challenges, particularly in medical settings where high-quality images and annotations are essential for supervised training, validation, and testing of AI algorithms (Park and Han, 2018). The lack of diverse samples and limited sample sizes from different geographic areas impede the generalizability and accuracy of developed solutions (Soffer et al., 2019). Acquiring medical image datasets for ML training purposes is a difficulty faced by many research groups due to the scarcity and the challenges involved in acquiring and preparing the images. Moreover, accessing appropriate clinical installations with expensive medical devices and the subsequent tasks of curating, anonymizing, analyzing, and annotating clinical data can be costly and time-consuming (Langlotz et al., 2019). Additionally, even in the case of open-source datasets, manual inspection of each image becomes necessary as some images may contain free-form annotations that cannot be reliably removed using automated methods (Willemink et al., 2020). Henceforth, publicly available well-curated annotated medical imaging datasets with high-quality ground truth pathological labels remain limited (National Lung Screening Trial Research Team, 2011; Clark et al., 2013; Sudlow et al., 2015; Wang et al., 2017; Bycroft et al., 2018; Mei et al., 2022).

When working with limited datasets, the issue of overfitting becomes particularly concerning. To address this challenge, researchers have proposed lightweight models that can still extract essential features (Sarvamangala and Kulkarni, 2022). However, it is important to note that the effectiveness of different models also depends on the imaging modality. For instance, Morid et al. (2021) suggest that deep models may be more suitable for X-ray, endoscopic, and ultrasound images, while shallow models could be optimal for processing OCT and photography of skin lesions and fundus images. However, in situations where gathering millions of training images is impractical, researchers have proposed alternative techniques such as transfer learning and data augmentation (Shorten and Khoshgoftaar, 2019) and transfer learning (Pan and Yang, 2009).

Transfer learning has proven to be effective in medical imaging, with many models pre-trained on non-medical imaging datasets successfully applied to real-world medical datasets (Xie and Richmond, 2018; Parakh et al., 2019; Ghesu et al., 2022). However, there are differing opinions on the optimal approach. For instance, Yu et al. (2019) found that retraining models from scratch achieve the highest diagnostic accuracy. These varying perspectives could be attributed to the diversity of data subjects and imaging modalities. Nonetheless, a comprehensive investigation of the characteristics of medical data and the application of transfer learning with CNN models is still lacking (Kim et al., 2022). Furthermore, it is crucial to note that successful transfer learning requires a reasonably large sample size, diverse images, and similarity between the training and target application images (Cheplygina et al., 2019). Data augmentation, another technique used in medical imaging, presents specific challenges. Firstly, it requires a careful selection of appropriate transformations for each modality and anatomy. Secondly, manual verification is necessary to ensure that the transformations do not alter the image's label or any relevant information. Moreover, it is important to consider that highly augmented data may cause the training data to deviate significantly from the testing data (Shorten and Khoshgoftaar, 2019).

Deep learning researchers must prioritize the development of methods that can achieve good performance even with small datasets, without the need to use data augmentation or transfer learning because both approaches may bias clinical diagnosis. In this regard, we propose a compact morphological-convolutional neural network (MCNN) for medical image diagnosis, which is trained using an extreme learning machine (ELM) and leverages the potential of Random Forest (RF) to fully explore the features created by the novel architecture. Unlike deep structures that require large amounts of data for effective training, MCNN adopts a more efficient design by incorporating mathematical morphology to identify important non-linear features in specific medical cases. We evaluated the effectiveness of our method by applying it to two common medical conditions glaucoma and melanoma. The structure of this article is organized as follows: Section 2 presents the methodology and datasets employed, Section 3 highlights the obtained results, Section 4 discusses our findings, and finally, Section 5 presents our conclusion.

## 2. Materials and methods

### 2.1. Architecture

**Figure 1:**
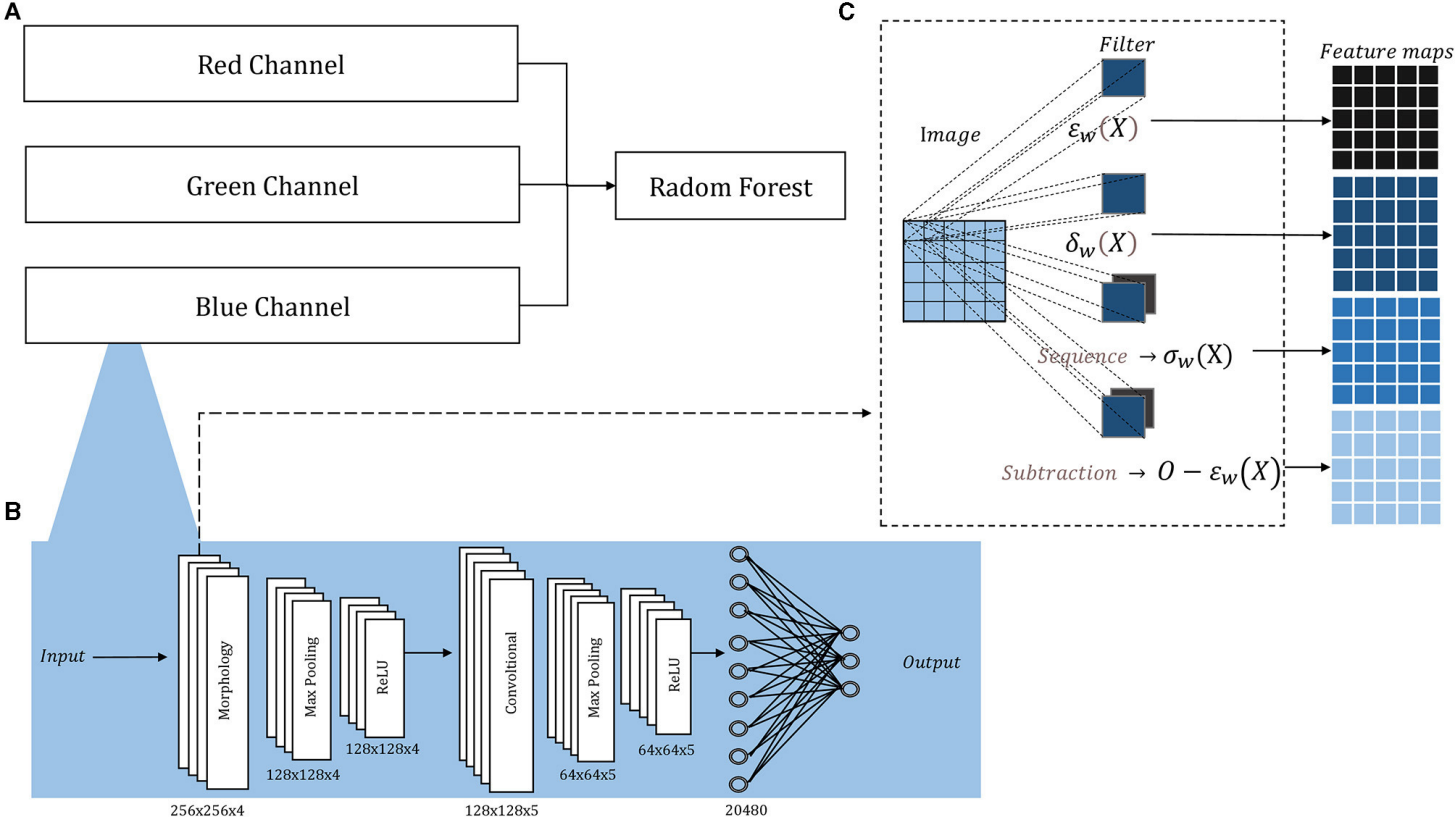
Architecture overview: **(A)** The probability outputs (a neural network per channel) are the inputs of a Random Forest classifier; **(B)** the detailed architecture of each neural network, the sized annotated below each layer corresponded to both ORIGA and ISIC images; **(C)** inside the morphological layer, four operations are parallelly applied.

Figure 1 depicts our compact model designed to overcome the limitations posed by the lack of large training sets in medical diagnosis. The morphological-convolutional neural network (MCNN) combines the power of convolutional and morphological operations within its architecture. In this model, three independent neural networks are trained, and their output probabilities are then used as inputs for a Random Forest (RF) classifier (Ho, 1995) (Figure 1A). Each neural network focuses on extracting features from a specific color channel and is trained using the Extreme Learning Machine (ELM) algorithm (Huang et al., 2004).

Morphological operations applied to 2D images are extensively documented (Serra, 1986, 2020; Serra and Soille, 2012; Najman and Talbot, 2013), for both binary and grayscale images. Their implementation is fast, straightforward, and comes with a minimal computational cost. Therefore, it was decided to use independent neural networks to handle 2D images, avoiding the complexity of direct 3D morphological operations.

The architecture of each neural network resembles a basic CNN, consisting of convolutional layers followed by Max-Pooling and ReLU activation, and concluding with a fully connected layer. The convolutional and morphological layers were performed with a stride of 1, *same* padding, and four filters in the first layer and five in the second, in both cases the filters sizes were 5 × 5 pixels the small number. Figure 1B illustrates this architecture, showcasing its adaptability to medical classification tasks, while Supplementary Figure 1 provides the architecture for basic classification tasks.

Additionally, we included a morphological layer in which we considered the following operations – erosion, dilation, opening, closing, and morphological residual – for grayscale images:


(1) $$\begin{array}{lll} \varepsilon_{w}\left(X\right) & = & \;\overset{n}{\underset{i=1}{\wedge }}\left(x_{i}\;*\;w_{i}\right)\;=\;X\;⊝\;w, \end{array}$$

(2) $$\begin{array}{lll} \delta_{w}\left(X\right) & = & \;\overset{n}{\underset{i=1}{\vee }}\left(x_{i}\;*\;w_{i}\right)=\;X\;⊕\;w, \end{array}$$

(3) $$\begin{array}{lll} \sigma_{w}\left(X\right) & = & \;\left(X\;⊝\;w\right)⊕\;w, \end{array}$$

(4) $$\begin{array}{lll} \gamma_{w}\left(X\right) & = & \;\left(X\;⊕\;w\right)⊝\;w, \end{array}$$

(5) $$\begin{array}{l} \mu_{w}=\;O-\;\alpha ,where\\ \;\alpha \;\in \left\{\varepsilon_{w}\left(X\right),\;\delta_{w}\left(X\right),\sigma_{w}\left(X\right),\gamma_{w}\left(X\right)\right\}, \end{array}$$

and *X* and *w* correspond to the image and the filter, respectively, being *x*_*i*_ the *i* − *pixel* of the image in the window, *n* the number of elements in the filter, and *O* the original image.

These operations are all included within a single layer, where they are applied in parallel. In conventional convolutional layers, the same operation (convolution) is applied multiple times using various filters, leading to distinct feature maps generated from the same input image. However, in The morphological layer, a varied assortment of operations is applied to the same input image. Consequently, the resulting output feature maps arise from not only diverse filters but also distinct operations. The layer holds a sequence operation, which allows for the sequential application of operations, resulting in the creation of openings and closings, and a subtraction operation, which allows us to create the morphological residual by subtracting a morphological operation from the original image. A different filter is learned per operation. Within this layer, the weights, which undergo an updating process similar to that employed for the convolutional layer, are binarized. The binarization involves applying a threshold derived from the midpoint between the normalization range's two extremes. In Figure 1C, we depict the internal configuration of the morphological layer.

### 2.2. Datasets

**Figure 2:**
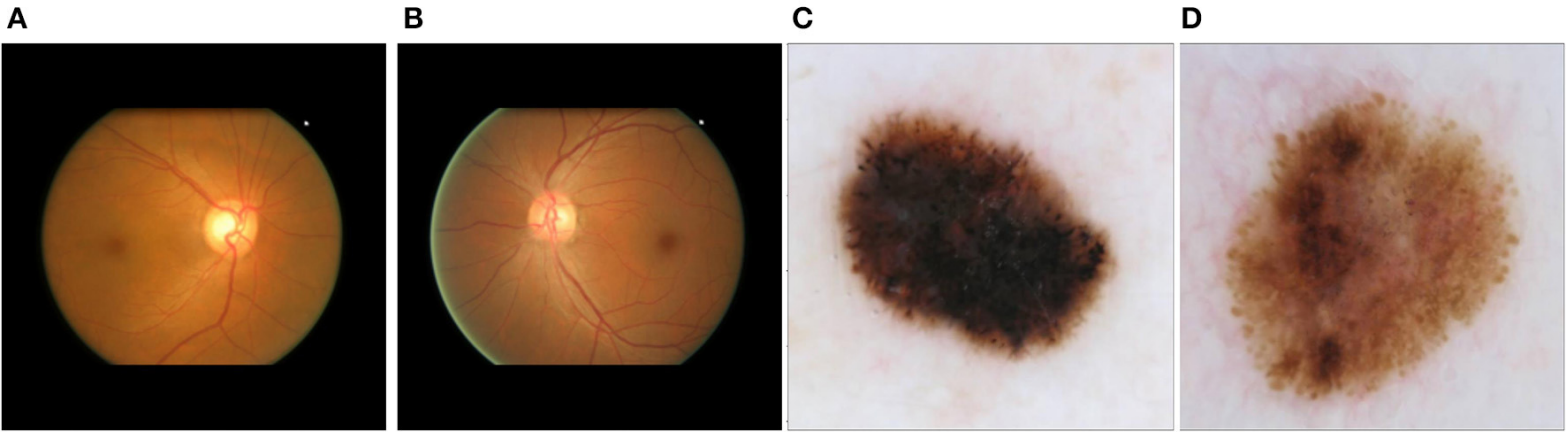
Main datasets for the study: **(A)** fundus image corresponding to glaucoma; **(B)** fundus image corresponding to a non-glaucoma eye disease; **(C)** dermoscopic image corresponding to melanoma; **(D)** dermoscopic image corresponding to non-melanoma skin tumor (melanocytic nevus).

To assess the performance of our proposed method for disease detection, we selected a simple classification task to ensure the proper functioning of the method, as well as two medical classification tasks where the shape of certain elements in the image plays a crucial role in disease identification. The first dataset used was the German Traffic Sign Recognition Benchmark (GTSRB) (Houben et al., 2013). Examples of the two classes can be seen in Supplementary Figures 2A, B. The second dataset employed was the Online Retinal Fundus Image Database for Glaucoma Analysis and Research (ORIGA-light) (Zhang et al., 2010) (Figures 2A, B). His dataset comprises 650 fundus images, with 168 representing glaucoma cases and 482 representing non-glaucoma cases. The images were meticulously annotated by trained professionals. The original image size is 2048 × 3072 pixels, but for our study, they were resized to 256 × 256 pixels. The third dataset was obtained from the International Skin Imaging Collaboration (2020) contribution (Figures 2C, D). It consists of 4,522 images of malignant tumors (melanoma) and 20,809 images of benign lesions (actinic keratosis, basal cell carcinoma, benign keratosis, dermatofibroma, melanocytic nevus, squamous cell carcinoma, and vascular lesion). The dataset includes 11,661 females, 13,286 males, and 384 individuals of unknown gender, covering an age range of 0 to 85 years. The images were captured from various body parts such as the anterior torso, head, neck, lateral torso, lower extremity, oral, genital, palms, soles, posterior torso, and upper extremity. For this study, we randomly selected 1,000 images, consisting of 500 malignant and 500 benign cases. These images were resized to 256 x 256 pixels.

### 2.3. Experimental procedures

Since this hybrid approach comprises two components—neural networks and a traditional machine learning method—a comparative analysis was conducted between the selected Random Forest and three alternative classifiers: Gaussian Naïve Bayes, Support Vector Machine (SVM), and AdaBoost. This experiment enables us to elucidate the rationale behind incorporating the Random Forest as the concluding component of the methodology.

The performance evaluation of the Morphological-Convolutional Neural Network (MCNN) involved two experimental procedures: an internal evaluation to ensure the proper functioning of the method and an external evaluation to assess its performance on small datasets. For the internal evaluation, two aspects were assessed using two datasets. First, the stability of the method was measured by testing it with 10 different seeds to observe the variability resulting from weight initialization and the Random Forest (RF) dependence on randomness. Second, the contribution of each channel-individual neural network to the complete architecture was examined. The accuracy per channel was compared with the accuracy of the complete architecture using all three channels together and a random forest classifier. This comparison was performed using 30 different test splits, and the median of these runs was considered the main result for further comparisons.

In the medical diagnostic evaluation, the two medical tasks were compared against common CNN architectures: ResNet-18, ShuffleNet-V2, and MobileNet-V2. Due to the limited dataset size, architectures with the fewest parameters were chosen. Two approaches were followed with these models: full training from random weights and transfer learning. For the transfer learning approach, models pre-trained with ImageNet (Deng et al., 2009). ImageNet is a dataset available in frameworks such as Pytorch and Tensorflow, making it easy to handle. Furthermore, previous studies have demonstrated that the gains in accuracy of pre-training with a medical dataset, compared to ImageNet, do not outweigh the time consumed on this task (Morid et al., 2021). The pre-trained weights were frozen, except for the final fully connected layer, which was replaced with a new layer having random weights. Only this layer was trained. Before comparing the models, we inspected the validation losses and accuracies for both training approaches, ensuring the correctness of the settings. The performance of the CNNs on the easy classification task (GTSRB dataset) served as the benchmark.

**Figure 3:**
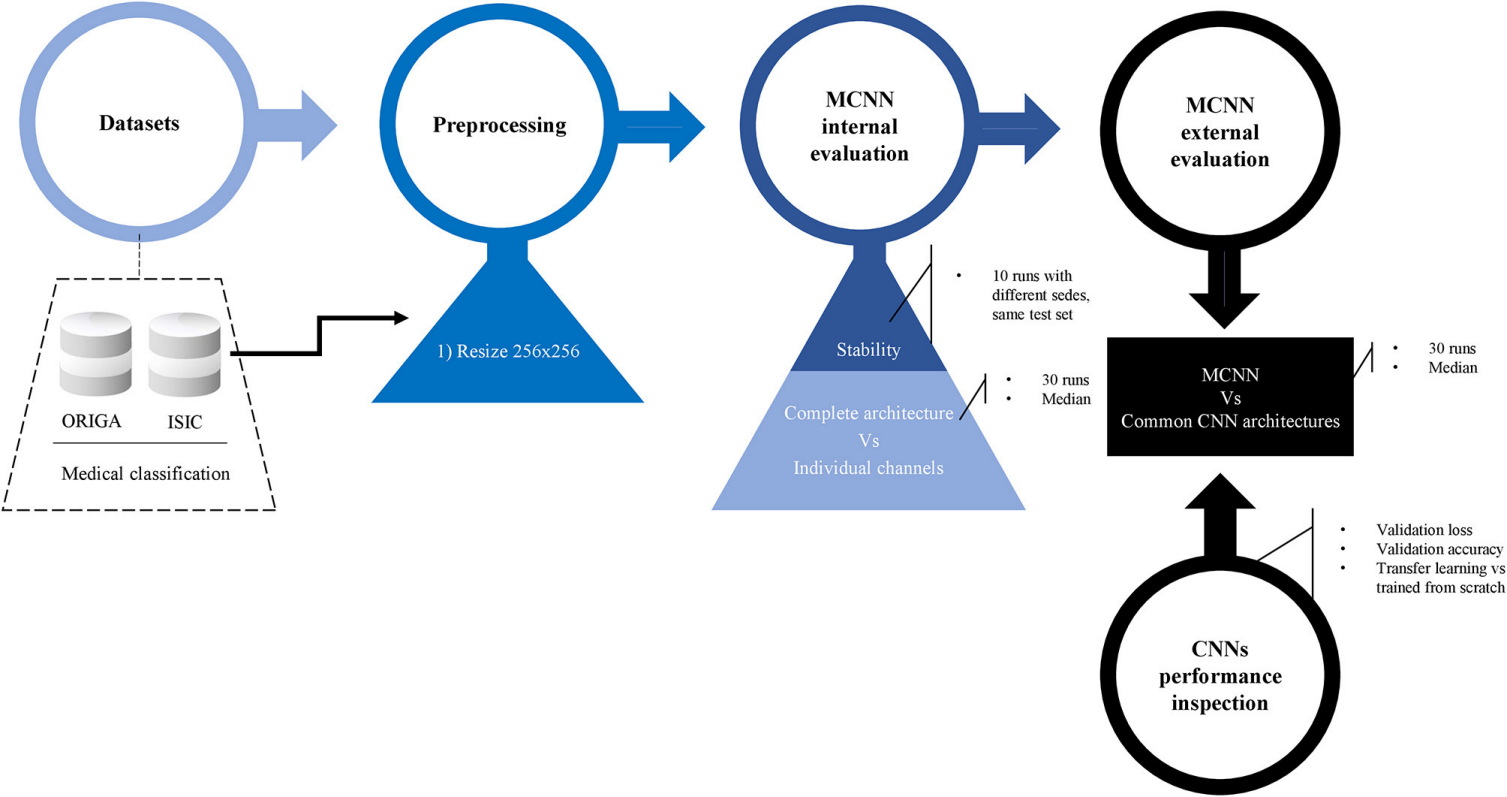
Methodology. Preprocessing details, internal evaluation using two experiments, and external evaluation against other common CNN architecture. Before the final comparison, CNN's performances were explored in detail. Two classification tasks were considered: glaucoma and melanoma.

For a summary of the methodology, refer to Figure 3.

### 2.4. Classification specifications

For the classification tasks using the MCNN method, the datasets was divided into 80% for training and 20% for testing, using a stratified split. As the optimizer used in this case is a classic ELM, a validation set is not required. The weights were randomly initialized using the method described in He et al. (2016). In ELM, the only parameter requiring tuning is a regularization parameter, which was set as specified in Supplementary Table 1. For the experiment of stability, the method was executed 10 times with different seeds but the same test set was kept, and for the rest of the experiments the method was executed 30 times exploring different test sets and proceeding to obtain the median of each classification metric.

In this study, the neural networks were structured with two layers (Figure 1B): an initial morphological layer followed by a convolutional layer. Within the morphological layer, parallel operations included an erosion, a dilation, an opening, and a residual operation consisting of subtracting an erosion from the original image. For each of these operations, distinct 5 × 5 filters were applied – each tailored to a specific operation – yielding four different feature maps from a single original image (Figure 1C). The convolutional layer was applied with 5 L of 5 × 5.

The common three models were divided into 60, 20, and 20% for training, validation, and testing sets, respectively, again using a stratified split. The loss function used was the binary cross-entropy loss, with stochastic gradient descent as the optimizer and a learning rate of 0.01, gamma of 0.9, 24 epochs, and a momentum of 0.9; but the ShuffleNet-V2 that was trained from scratch for glaucoma classification required a momentum of 0.1.

The resizing of the images and the classification tasks were performed using the Pytorch framework (Paszke et al., 2019) with Python (Python Software Foundation. Python Language Reference, version 3.7.14. Available at http://www.python.org) (Van Rossum and Drake, 1995). Figure 3 shows a summary of the methodology followed in this study.

## 3. Results

### 3.1. Classifiers comparison

Here, we present the outcomes of the comparison among the four machine learning classifiers. Table 1 provides a comprehensive overview of the resulting classification metrics.

**Table 1:** Classification results using different machine learning methods as final step in the hybrid MCNN.

**Classifier**	**Accuracy**	**Balanced accuracy**	**AUC**	**Error**
	**95% CI**	**95% CI**	**95% CI**	**95% CI**
**ORIGA**				
Random Forest	0.73 (0.66, 0.79)	0.57 (0.50, 0.64)	0.65 (0.53, 0.74)	0.27 (0.24, 0.35)
Naïve Bayes	0.69 (0.62, 0.75)	0.55 (0.48, 0.62)	0.64 (0.55, 0.73)	0.30 (0.24, 0.37)
SVM	0.672 (0.60, 0.73)	0.51 (0.44, 0.58)	0.56 (0.46, 0.66)	0.32 (0.26, 0.39)
AdaBoost	0.66 (0.60, 0.73)	0.56 (0.49, 0.62)	0.52 (0.43, 0.62)	0.33 (0.26, 0.39)
**ISIC**				
Random Forest	0.88 (0.84, 0.92)	0.88 (0.84, 0.91)	0.94 (0.91, 0.97)	0.11 (0.11, 0.17)
Naïve Bayes	0.84 (0.80, 0.88)	0.84 (0.80, 0.88)	0.90 (0.87, 0.94)	0.15 (0.11, 0.19)
SVM	0.86 (0.82, 0.90)	0.86 (0.82, 0.90)	0.93 (0.90, 0.96)	0.13 (0.09, 0.17)
AdaBoost	0.86 (0.82, 0.89)	0.85 (0.82, 0.89)	0.93 (0.90, 0.96)	0.14 (0.10, 0.17)

### 3.2. Stability

**Figure 4:**
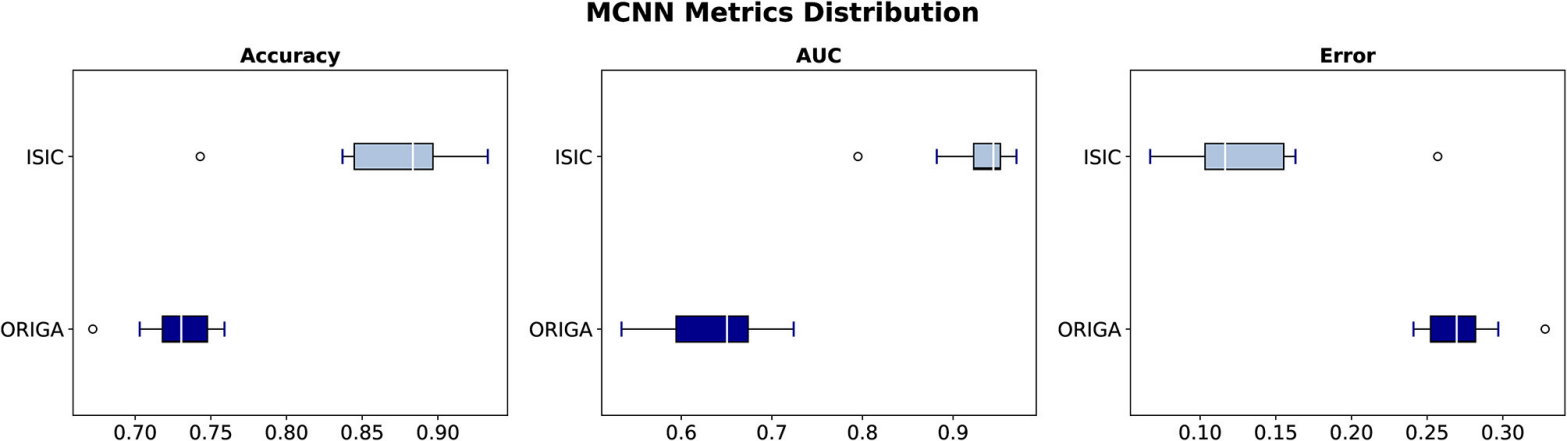
Classification metrics distribution through 10 seeds with ORIGA and ISIC datasets.

**Figure 5:**
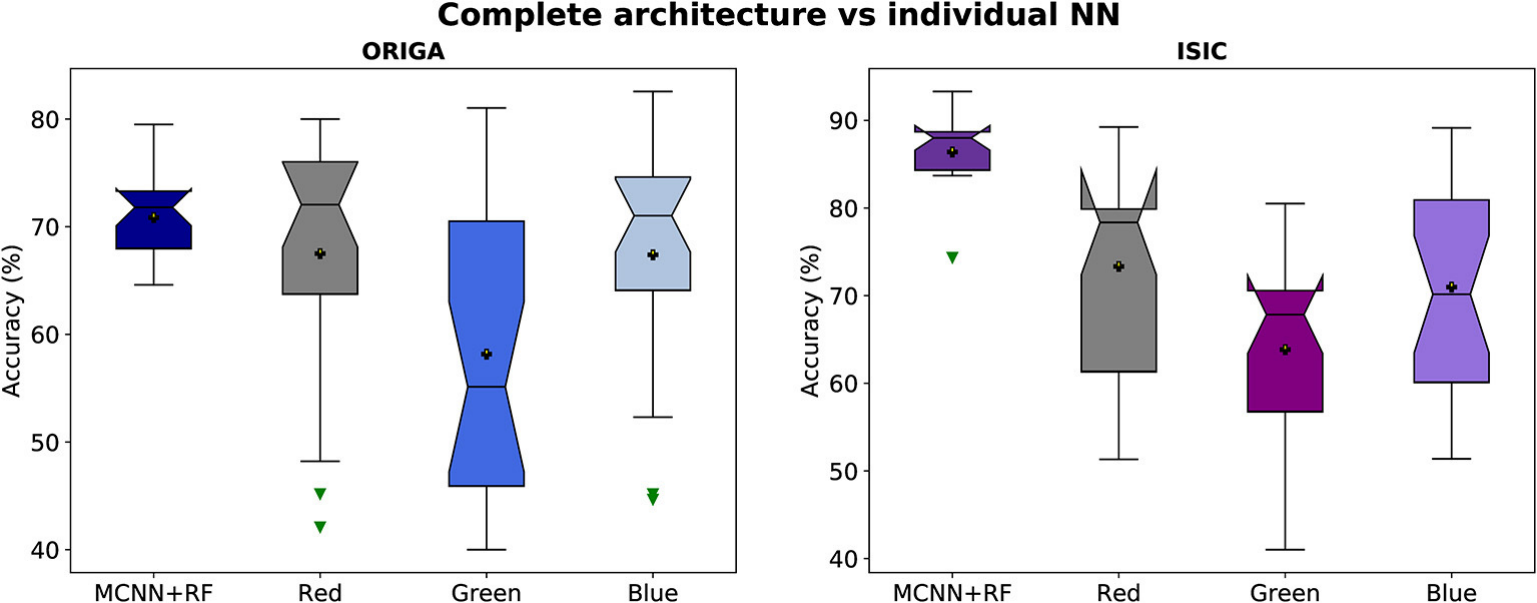
Accuracy distributions of two classification tasks (glaucoma, and melanoma). Complete architecture (MCNN + Random Forest) vs. neural networks working with each channel (Red, Green, and Blue).

The stability of the method is explored by looking at the distribution of different classification metrics through 10 different seeds with the different datasets used in this study (Figure 4, Supplementary Figure 3). In Table 2 a statistical summary of this experiment is found. Figure 5 presents the classification performance by channel against the result from the complete architecture.

**Table 2:** Statistical results from the 10 runs per dataset.

**Statistical metric**	**Accuracy**	**Balanced accuracy**	**AUC**	**Error**
**ORIGA**				
Mean	0.6673	0.6581	0.7026	0.3327
Variance	0.0010	0.0009	0.0003	0.0010
Std	0.0322	0.0302	0.0196	0.0322
Median	0.6670	0.6640	0.7035	0.3330
**ISIC**				
Mean	0.8660	0.8657	0.9250	0.1340
Variance	0.0027	0.0027	0.0027	0.0027
Std	0.0523	0.0523	0.0521	0.0523
Median	0.8835	0.8830	0.9445	0.1165

### 3.3. CNNs performance

**Figure 6:**
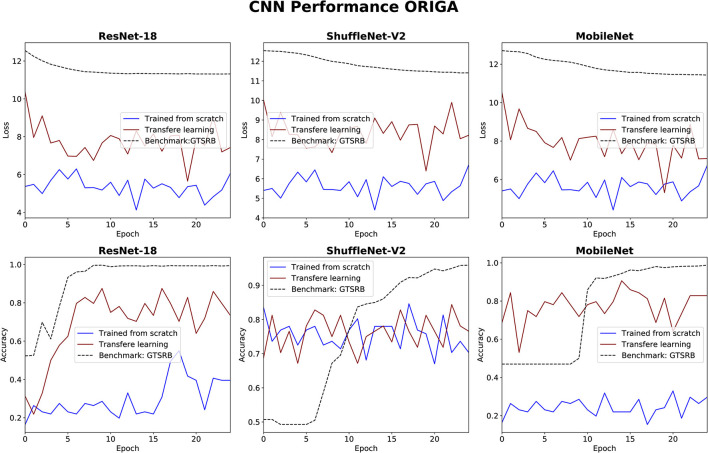
Results for glaucoma classification using three CNNs. The GTSRB classification is used as benchmark.

**Figure 7:**
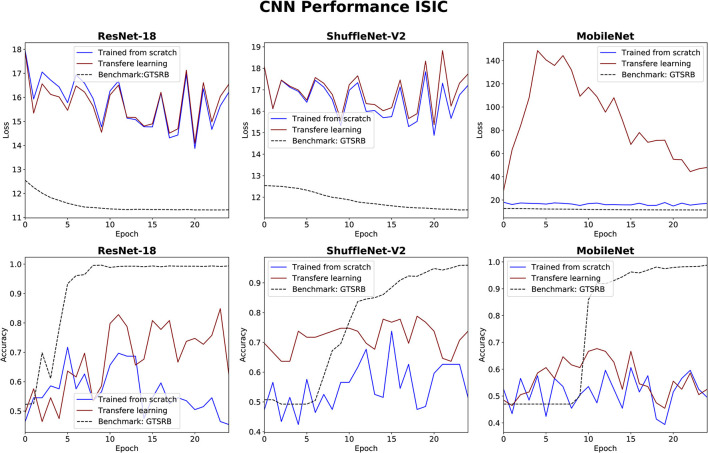
Results for melanoma classification using three CNNs. The GTSRB classification is used as benchmark.

The performance of the three CNN methods in medical classifications was evaluated by observing their validation losses, and accuracies. The easy classification task (GTSRB) was used as unit test to ensure that the settings were appropriate. Figures 6, 7 show results for glaucoma and melanoma classification, respectively.

### 3.4. Medical diagnosis evaluation

**Figure 8:**
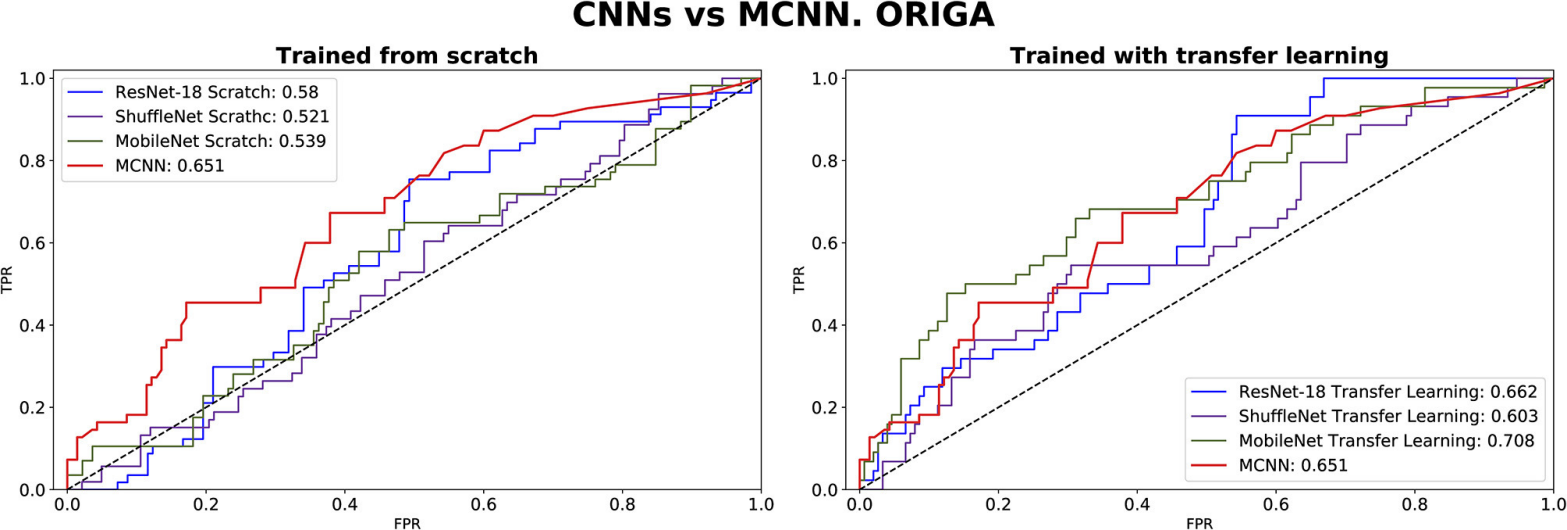
ROC-AUC of three common CNNs and of the MCNN method for glaucoma classification trained from scratch **(left)** and using transfer learning **(right)**.

**Figure 9:**
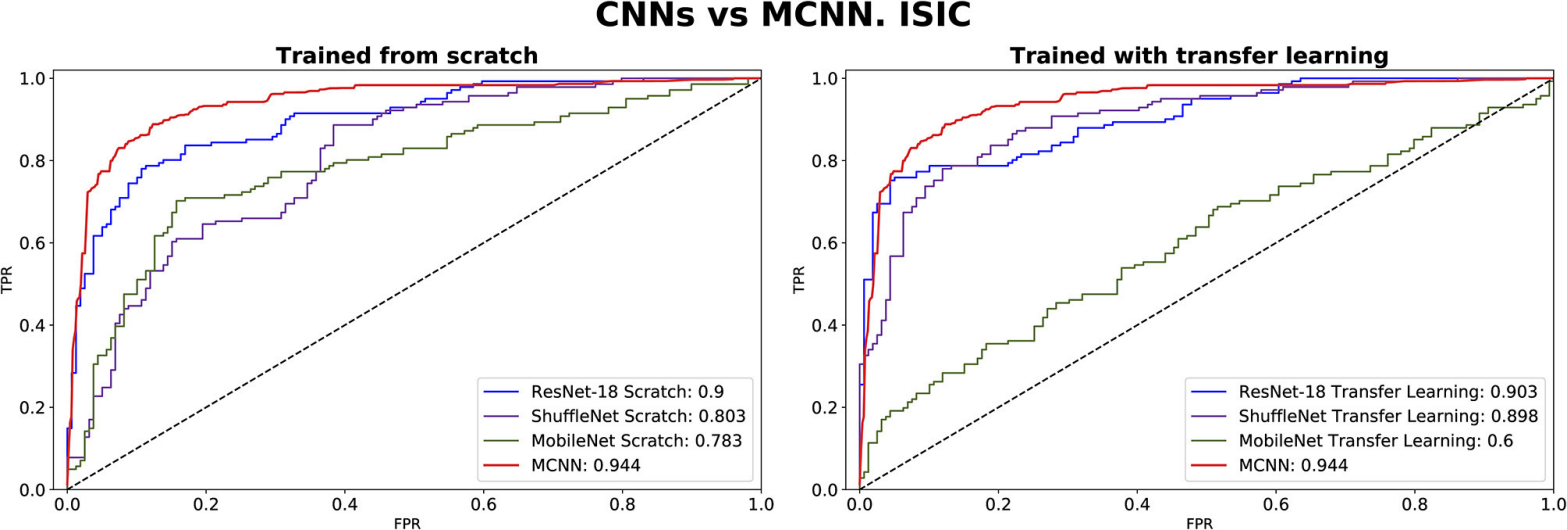
ROC-AUC of three common CNNs and of the MCNN method for melanoma classification trained from scratch **(left)** and using transfer learning **(right)**.

The comparison between our MCNN method and the three selected CNN architectures is presented in Figures 8, 9 for glaucoma and melanoma, respectively. It includes the ROC-AUCs for both training processes: from scratch and with transfer learning. The complete metrics, such as balanced accuracy, error, AUC, and the number of parameters, for both ORIGA and ISIC datasets, are presented in Tables 3, 4, respectively.

**Table 3:** Results of glaucoma classification using the ORIGA dataset and number of parameters trained per method.

**Model**	**Accuracy**	**Balanced accuracy**	**AUC**	**Error**	**No. of params**
	**95% CI**	**95% CI**	**95% CI**	**95% CI**	
ResNet-18 S	0.29 (0.23, 0.36)	0.49 (0.42, 0.56)	0.58 (0.49, 0.66)	0.70 (0.63, 0.76)	1.1 × 10^7^
ShuffleNet-V2 S	0.72 (0.66, 0.79)	0.50 (0.43, 0.57)	0.52 (0.43, 0.60)	0.27 (0.20, 0.33)	6.1 × 10^7^
MobileNet-V2 S	0.29 (0.22, 0.35)	0.50 (0.43, 0.57)	0.53 (0.45, 0.62)	0.70 (0.64, 0.77)	1.3 × 10^8^
ResNet-18 TL	0.76 (0.70, 0.82)	0.50 (0.43, 0.58)	0.66 (0.58, 0.74)	0.23 (0.17, 0.29)	513
ShuffleNet-V2 TL	0.73 (0.67, 0.80)	0.51 (0.44, 0.58)	0.60 (0.50, 0.69)	0.26 (0.20, 0.32)	1,025
MobileNet TL	0.78 (0.72, 0.84)	0.53 (0.46, 0.60	0.70 (0.61, 0.79)	0.21 (0.15, 0.27)	1,001
MCNN	0.73 (0.66, 0.79)	0.57 (0.50, 0.64)	0.65 (0.53, 0.74)	0.27 (0.24, 0.35)	6.1 × 10^4^

S stands for training from scratch and TL for training using transfer learning.

**Table 4:** Results of melanoma classification using the ISIC dataset and number of parameters trained per method.

**Model**	**Accuracy**	**Balanced accuracy**	**AUC**	**Error**	**No. of params**
	**95% CI**	**95% CI**	**95% CI**	**95% CI**	
ResNet-18 S	0.53 (0.48, 0.59)	0.50 (0.45, 0.56)	0.90 (0.86, 0.96)	0.46 (0.40, 0.52)	1.1 × 10^7^
ShuffleNet-V2 S	0.59 (0.53, 0.64)	0.57 (0.51, 0.62)	0.80 (0.75, 0.85)	0.40 (0.35, 0.46)	6.1 × 10^7^
MobileNet-V2 S	0.54 (0.48, 0.80)	0.51 (0.45, 0.57)	0.78 (0.72, 0.83)	0.54 (0.40, 0.51)	1.3 × 10^8^
ResNet-18 TL	0.76 (0.71, 0.80)	0.74 (0.69, 0.79)	0.90 (0.86, 0.93)	0.24 (0.19, 0.28)	513
ShuffleNet-V2 TL	0.68 (0.63, 0.73)	0.69 (0.64, 0.75)	0.89 (0.86, 0.93)	0.31 (0.26, 0.36)	1,025
MobileNet TL	0.45 (0.39, 0.51)	0.48 (0.42, 0.53)	0.60 (0.53, 0.66)	0.54 (0.49, 0.60)	1,001
MCNN	0.88 (0.84, 0.92)	0.88 (0.84, 0.91)	0.94 (0.91, 0.97)	0.11 (0.11, 0.17)	6.1 × 10^4^

S stands for training from scratch and TL for training using transfer learning.

## 4. Discussion

This study introduced the hybrid Morphological-Convolutional Neural Network (MCNN), which combines mathematical morphology operations with conventional convolutional layers as feature extraction layers. Our results provided a more comprehensive analysis compared to our preliminary findings, clearly demonstrating the superiority of the hybrid deep learning method over standard CNN architectures (Canales-Fiscal et al., 2023). The addition of a Random Forest in the final part of the method is justified by observing at the performance of the different classifiers in Table 1. Although Random Forest was not statistically significant higher in most of the cases, it is still the best method performing in both classification tasks, with an AUC score of 0.65 (0.53, 0.74) 95% CI for the glaucoma classification followed by Naïve Bayes with 0.64 (0.55, 0.73) 95% CI, and with 0.94 (0.91, 0.97) 95% CI in the melanoma classification followed by SVM with 0.93 (0.90, 0.96) 95% CI.

To assess the model's performance, evaluations using different seeds were conducted, as depicted in Figure 4. The results indicate that the MCNN exhibits higher stability in medical classifications, with variances of <0.001 for the ORIGA dataset and ≤0.003 for the ISIC dataset. While a single outlier was observed in both glaucoma and melanoma classifications, the distribution of classification results for the ORIGA dataset tends to be smaller than that of the ISIC dataset (Figure 4). It is important to note that in both cases, 50% of the data showed variations of < 0.05 across all metrics (Figure 4).

In Figure 5, it is evident that the complete architecture of the MCNN demonstrates both stability and improved results compared to the individual neural networks (NNs). It is observed that the individual NNs occasionally yield random classifications. Specifically, when considering the ORIGA dataset, the NN operating with the green channel exhibits a significant variation range (~23%) in 50% of the cases, indicating that this channel alone may not provide sufficient information for accurate classification. Moreover, the individual NNs consistently exhibit distributions with variations of 10% or more in accuracy. In contrast, the complete architecture remains stable, with variations of ~5% or less. The mean values of the individual NNs are consistently smaller than those of the complete architecture, as expected since using separate channels limits the available information. For the ORIGA dataset, the NNs utilizing the red and blue channels show mean results closer to the mean of the complete architecture. While there are a few instances where the individual NNs outperform the complete architecture in terms of accuracy, these cases are not substantial enough to be considered representative. A similar trend was observed with the ISIC dataset, although in this case, it was identified the presence of skewed data that affected performance.

The performance of the CNN architectures (ResNet-18, ShuffleNet-V2, and MobileNet-V2) is worth discussing. Figures 6, 7 clearly illustrate that their poor performance is primarily attributed to the small size of the datasets rather than incorrect settings. This assertion is supported by the fact that neither of the methods, when applied to the ISIC and ORIGA datasets, achieved satisfactory convergence, resulting in oscillating validation accuracy. Conversely, when utilizing the GTSRB dataset as a benchmark, the loss demonstrates convergence and the validation accuracy steadily increases, aligning with our expectations.

In comparing our method, MCNN, with other CNN architectures, three key observations were made. Firstly, when trained from scratch, MCNN outperforms CNNs, albeit only slightly. Figure 8 illustrates this, where MCNN achieved an AUC of 0.651 with the ORIGA dataset, while the best CNN method (MobileNet-V2) achieved an AUC of 0.539. However, upon closer examination of the confidence intervals in Table 3, it is found that neither method demonstrates statistically significant superiority. When considering accuracy, MCNN performs with 0.73 (0.66, 0.79) 95% CI, while ResNet-18 and MobileNet-V2 fail to classify at all. Balanced accuracy further confirms this trend, as the CNN methods trained from scratch provide random classification, whereas MCNN yields a lower but non-random classification with a higher confidence interval of 0.57 (0.50, 0.64). Additionally, it is important to note that MCNN requires fewer parameters for training compared to CNN methods trained from scratch. Figure 9 highlights that, with the ISIC dataset, MCNN achieves the best AUC (0.944), surpassing the CNN methods when trained from scratch (0.9 for ResNet-18 as the second best). However, from Table 3, it becomes evident that MCNN is statistically significantly better only than ShuffleNet-V2 and MobileNet-V2, with confidence intervals of 0.94 (0.91, 0.97) and 0.8 (0.75, 0.86), respectively. MCNN's performance overlaps with that of ResNet-18, with confidence intervals of 0.95 (0.91, 0.97) for MCNN and 0.90 (0.86, 0.96) for ResNet-18. Lastly, when examining accuracy in Table 4, our method consistently outperforms the others, with an accuracy of 0.88 (0.84, 0.91) 95% CI, while the closest result was observed for ShuffleNet-V2 with an accuracy of 0.57 (0.51, 0.62) 95% CI.

The second observation made was that MCNN performed comparably to CNN methods trained using transfer learning. When considering the classification results with the ORIGA dataset, the AUC of the MCNN method (Figure 8) falls within the same range as the CNN methods, and none of the methods exhibit statistically significant differences. This pattern was also evident in the accuracy results presented in Table 3. Turning to the ISIC dataset, our method achieved a higher AUC compared to CNN methods trained using transfer learning, with an AUC of 0.944, whereas ResNet-18 attained 0.903 as the second-best performance. However, upon reviewing Table 4, we discovered that MCNN was only statistically significantly superior to MobileNet-V2, with a confidence interval of 0.94 (0.91, 0.97), while MobileNet-V2 had a confidence interval of 0.60 (0.53, 0.66).

The third observation highlights that MCNN exhibited better performance on imbalanced datasets. When comparing the change from accuracy to balanced accuracy (Table 3), it is observed that the MCNN method shows a smaller change, with a difference of 0.16, whereas ShuffleNet-V2 had the second smallest change of 0.22. Furthermore, in the final experiment, it was found that our method performed more effectively with the ISIC dataset than with the ORIGA dataset. When considering the accuracy of MCNN, it outperformed all other methods, regardless of the training approach, with the ISIC dataset. Specifically, the accuracy of MCNN was 0.88 (0.84, 0.91) with a 95% confidence interval, while the second-best result was obtained by ResNet-18 trained using transfer learning, with an accuracy of 0.74 (0.69, 0.79) (Table 3). However, when examining the results with the ORIGA dataset, the performance of MCNN was within the range of the methods trained using transfer learning (Table 3).

This study had some limitations that should be considered. Firstly, the resizing of images to 256 × 256 pixels may have affected the classification performance, as vital information might have been lost during the process. Secondly, the capability of the morphological layer requires further exploration. Each medical case could benefit from a specific combination of morphological operations. In this study, all operations were activated simultaneously, and a single morphological layer was used. Future work is required to investigate this aspect more comprehensively. Additionally, there were limitations in terms of computational resources. The ELM optimizer, although efficient and effective, requires a higher amount of memory compared to other optimizers, which posed limitations on its usage. Moreover, training CNNs from scratch proved to be time-consuming, with each run taking up to 24 h. As a result, we reduced the number of samples of the ISIC and GTSRB datasets. Furthermore, it is important to note that our method did not achieve the state-of-the-art accuracy and AUC for glaucoma classification with the ORIGA dataset, which is reported as 78.32% and 0.874, respectively (Bajwa et al., 2019; Elangovan and Nath, 2021). However, it is worth considering that the common approach in the literature for glaucoma classification involves segmenting the optic disk (Sengupta et al., 2020), whereas, in this study, complete fundus images were utilized. Given that the necessary information for detecting glaucoma is exclusively contained within the optic disk, there is a suspicion that the additional information present in the remaining portion of the image might be affecting the training process. Additionally, the extreme resizing –going from 2048 × 3072 to 256 × 256 – influences the final outcome. Upon examining the classification results from the other CNN methods in Table 3, it becomes apparent that the outcomes fall within a similar range as those achieved by the MCNN method. The balanced accuracy spans from 0.49 (0.42, 0.56) 95% CI to 0.57 (0.50, 0.64) 95% CI, and the AUC score ranges from 0.52 (0.43, 0.60) 95% CI to 0.70 (0.61, 0.79) 95% CI. This leads to the conclusion that the poor performance stems more from inadequate dataset preprocessing rather than inherent limitations of the MCNN method. It would be interesting to explore the capability of MCNN for glaucoma classification using the optic disk segmentation approach and compare its performance accordingly. Lastly, although we opted for a simple architecture to manage small datasets, we suspect that optimizing the architecture specifically for each medical case would improve performance. Researchers should explore this aspect to gain a better understanding of the overall effectiveness of the method.

## 5. Conclusion

In this study, it was introduced a novel hybrid ML approach called morphological-convolutional neural network (MCNN) for medical image diagnosis. By incorporating the extreme learning machine (ELM) for optimization and a Random Forest (RF) as the final classifier, our method demonstrated enhanced diagnostic capabilities. The combination of ELM and RF with the RBG layers of the network eliminates the need for a deep structure and reduces the data requirements for effective training. To evaluate the performance of our MCNN method, we conducted experiments on two medical diagnosis tasks: glaucoma identification using the ORIGA dataset and melanoma identification using the ISIC dataset. As points of comparison, three widely used CNN architectures were included: ResNet-18, ShuffleNet-V2, and MobileNet-V2, utilizing full training and transfer learning approaches. Our findings revealed that the MCNN method surpassed the performance of conventional CNN architectures when trained from scratch. Moreover, when compared to methods trained using transfer learning on small datasets, the MCNN method achieved comparable results. These results highlight the effectiveness of our MCNN approach in addressing the challenges posed by medical image diagnosis tasks. By leveraging the advantages of morphological operations, the ELM optimizer, and the RF classifier, our MCNN method offers a promising avenue for accurate and efficient medical image analysis. Further research should explore the potential of optimizing the MCNN architecture for specific medical cases and investigate its applicability to other medical image diagnosis tasks.

## Data availability statement

Publicly available datasets were analyzed in this study. This data can be found at: (1) https://www.kaggle.com/datasets/arnavjain1/glaucoma-datasets (2) https://www.kaggle.com/datasets/cdeotte/jpeg-isic2019-512x512.

## Author contributions

MC-F and JT-P conceived of the presented idea. MC-F carried out the computation and experiments. JT-P encouraged MC-F to investigate the current problems with a medical image dataset availability and supervised the findings of this work. All authors contributed to the article and approved the submitted version.

## References

[B1] Bajwa M. N. Malik M. I. Siddiqui S. A. Dengel A. Shafait F. Neumeier W.. (2019). Two-stage framework for optic disc localization and glaucoma classification in retinal fundus images using deep learning. BMC Med. Inf. Decision Making 19, 1–16. 10.1186/s12911-019-0842-8 31315618 PMC6637616

[B2] Bhateja V. Nigam M. Bhadauria A. S. Arya A. Zhang E. Y. D. (2019). Human visual system based optimized mathematical morphology approach for enhancement of brain MR images. J. Amb. Int. Hum. Comput. 3, 1–9. 10.1007/s12652-019-01386-z

[B3] Bycroft C. Freeman C. Petkova D. Band G. Elliott L. T. Sharp K.. (2018). The UK Biobank resource with deep phenotyping and genomic data. Nature 562, 203–209. 10.1038/s41586-018-0579-z 30305743 PMC6786975

[B4] Canales-Fiscal M. R. Tamez-Peña J. G. Mudduluru S. (2023). Glaucoma classification using a morphological-convolutional neural network trained with extreme learning machine. Med. Imag. Comput. Aided Diag. 12465, 590–600. 10.1117/12.265402536868341

[B5] Cheplygina V. Bruijne D. Pluim M. (2019). Not-so-supervised: a survey of semi-supervised, multi-instance, and transfer learning in medical image analysis. Med. Imag. Anal. 54, 280–296. 10.1016/j.media.2019.03.009 30959445

[B6] Clark K. Vendt B. Smith K. Freymann J. Kirby J. Koppel P.. (2013). The cancer imaging archive (TCIA): maintaining and operating a public information repository. J. Dig. Imag. 26, 1045–1057. 10.1007/s10278-013-9622-7 23884657 PMC3824915

[B7] Deepak S. Ameer P. M. (2021). Automated categorization of brain tumor from mri using cnn features and svm. J. Amb. Int. Hum. Comput. 12, 8357–8369. 10.1007/s12652-020-02568-w

[B8] Deng J. Dong W. Socher R. Li L. J. Li K. Fei-Fei L.. (2009). “Imagenet: A large-scale hierarchical image database,” in 2009 IEEE Conference on Computer Vision and Pattern Recognition, Honolulu, HI: IEEE, 248–255.

[B9] Elangovan P. Nath M. K. (2021). Glaucoma assessment from color fundus images using convolutional neural network. Int. J. Imag. Syst. Technol. 31, 955–971. 10.1002/ima.2249431344328

[B10] Esteva A. Kuprel B. Novoa R. A. Ko J. Swetter S. M. Blau H. M.. (2017). Dermatologist-level classification of skin cancer with deep neural networks. Nature 542, 115–118. 10.1038/nature21056 28117445 PMC8382232

[B11] Franchi G. Fehri A. Yao A. (2020). Deep morphological networks. Pattern Recognit. 102, 107246. 10.1016/j.patcog.2020.107246

[B12] Garbe C. Amaral T. Peris K. Hauschild A. Arenberger P. Basset-Seguin N.. (2022). European consensus-based interdisciplinary guideline for melanoma. Part 1: diagnostics: Update 2022. Eur. J. Cancer 170, 236–255. 10.1016/j.ejca.2022.03.008 35570085

[B13] García-Floriano A. Ferreira-Santiago Á. Camacho-Nieto O. Yáñez-Márquez C. (2019). A machine learning approach to medical image classification: Detecting age-related macular degeneration in fundus images. Comput. Electr. Eng. 75, 218–229. 10.1016/j.compeleceng.2017.11.008

[B14] Ghesu F. C. Georgescu B. Mansoor A. Yoo Y. Neumann D. Patel P.. (2022). Self-Supervised Learning From 100 Million Medical Images. 36466078 10.1117/1.JMI.9.6.064503 PMC9710476

[B15] He K. Zhang X. Ren S. Sun J. (2016). “Deep residual learning for image recognition,” in Proceedings of the IEEE Conference on Computer Vision and Pattern Recognition, Honolulu, HI: IEEE, 770–778.

[B16] Ho T. K. (1995). Random decision forests. Proc. Int. Conf. Doc. Anal. Recognit. 1, 278–282.

[B17] Houben S. Stallkamp J. Salmen J. Schlipsing M. Igel C. (2013). “Detection of traffic signs in real-world images: The German Traffic Sign Detection Benchmark,” in The 2013 International Joint Conference on Neural Networks (IJCNN), Honolulu, HI: IEEE, 1–8.

[B18] Huang G. Liu Z. Van Der Maaten L. Weinberger K. Q. (2017). “Densely connected convolutional networks,” in 2017 IEEE Conference on Computer Vision and Pattern Recognition (CVPR), Honolulu, HI: IEEE.

[B19] Huang G. B. Zhu Q. Y. Siew C. K. (2004). Extreme learning machine: a new learning scheme of feedforward neural networks. IEEE 2, 985–990. 10.1109/IJCNN.2004.138006818991365

[B20] International Skin Imaging Collaboration (2020). ISIC-Archive. Available online at: https://www.isic-archive.com

[B21] Iqbal S. Khan T. M. Naveed K. Naqvi S. S. Nawaz S. J. (2022). Recent trends and advances in fundus image analysis: a review. Comput. Biol. Med. 24, 106277. 10.1016/j.compbiomed.2022.106277 36370579

[B22] Jia A. D. Li B. Z. Zhang C. C. (2020). Detection of cervical cancer cells based on strong feature CNN-SVM network. Neurocomputing 411, 112–127. 10.1016/j.neucom.2020.06.006

[B23] Khalifa N. E. M. Taha M. H. N. Hassanien A. E. Taha S. H. N. (2020). The detection of covid-19 in ct medical images: a deep learning approach. Big data analytics and artificial intelligence against COVID-19. Innov. Vision Appr. 78, 73–90. 10.1007/978-3-030-55258-9_5

[B24] Kim H. E. Cosa-Linan A. Santhanam N. Jannesari M. Maros M. E. Ganslandt T.. (2022). Transfer learning for medical image classification: a literature review. BMC Med. Imag. 22, 69. 10.1186/s12880-022-00793-7 35418051 PMC9007400

[B25] Langlotz C. P. Allen B. Erickson B. J. Kalpathy-Cramer J. Bigelow K. Cook T. S.. (2019). A roadmap for foundational research on artificial intelligence in medical imaging: from the 2018 NIH/RSNA/ACR/The academy workshop. Radiology 291, 781–791. 10.1148/radiol.2019190613 30990384 PMC6542624

[B26] Lee H. Yune S. Mansouri M. Kim M. Tajmir S. H. Guerrier C. E.. (2019). An explainable deep-learning algorithm for the detection of acute intracranial haemorrhage from small datasets. Nat. Biomed. Eng. 3, 173–182. 10.1038/s41551-018-0324-9 30948806

[B27] Ma N. Zhang X. Zheng H. T. Sun J. (2018). “Shufflenet v2: Practical guidelines for efficient CNN architecture design,” in Proceedings of the European Conference on Computer Vision (ECCV), Cham: ECCV, 116–131.

[B28] Mei X. Lee H. C. Diao K. Y. Huang M. Lin B. Liu C.. (2020). Artificial intelligence–enabled rapid diagnosis of patients with COVID-19. Nat. Med. 26, 1224–1228. 10.1038/s41591-020-0931-3 32427924 PMC7446729

[B29] Mei X. Liu Z. Robson P. M. Marinelli B. Huang M. Doshi A.. (2022). RadImageNet: an open radiologic deep learning research dataset for effective transfer learning. Radiology: Artif. Int. 4, e210315. 10.1148/ryai.210315 36204533 PMC9530758

[B30] Mellouli D. Hamdani T. M. Sanchez-Medina J. J. Ayed M. B. Alimi A. M. (2019). Morphological convolutional neural network architecture for digit recognition. IEEE Trans. Neural Netw. Learn. Syst. 30, 2876–2885. 10.1109/TNNLS.2018.2890334 30676985

[B31] Morid M. A. Borjali A. Del Fiol G. (2021). A scoping review of transfer learning research on medical image analysis using ImageNet. Comput. Biol. Med. 128, 104115. 10.1016/j.compbiomed.2020.104115 33227578

[B32] Najman L. Talbot H. (2013). Mathematical Morphology: From Theory to Applications. London: John Wiley and Sons.

[B33] National Lung Screening Trial Research Team (2011). Reduced lung-cancer mortality with low-dose computed tomographic screening. N. Eng. J. Med. 365, 395–409. 10.1056/NEJMoa1102873 21714641 PMC4356534

[B34] Pan S. J. Yang Q. (2009). A survey on transfer learning. IEEE Trans. Know. Data Eng. 22, 1345–1359. 10.1109/TKDE.2009.191

[B35] Parakh A. Lee H. Lee J. H. Eisner B. H. Sahani D. V. Do S.. (2019). Urinary stone detection on CT images using deep convolutional neural networks: evaluation of model performance and generalization. Radiol. Artif. Int. 1, e180066. 10.1148/ryai.2019180066 33937795 PMC8017404

[B36] Park S. H. Han K. (2018). Methodologic guide for evaluating clinical performance and effect of artificial intelligence technology for medical diagnosis and prediction. Radiology 286, 800–809. 10.1148/radiol.2017171920 29309734

[B37] Paszke A. Gross S. Massa F. Lerer A. Bradbury J. Chanan G.. (2019). Pytorch: An imperative style, high-performance deep learning library. Adv. Neural Inf. Proc. Syst. 32, 8024–8035. 10.48550/arXiv.1912.01703

[B38] Sandler M. Howard A. Zhu M. Zhmoginov A. Chen L. C. (2018). “Mobilenetv2: Inverted residuals and linear bottlenecks,” in Proceedings of the IEEE Conference on Computer Vision and Pattern Recognition, Honolulu, HI: IEEE, 4510–4520.

[B39] Sarvamangala D. R. Kulkarni R. V. (2022). Convolutional neural networks in medical image understanding: a survey. Evol. Int. 15, 1–22. 10.1007/s12065-020-00540-3 33425040 PMC7778711

[B40] Sengupta S. Singh A. Leopold H. A. Gulati T. Lakshminarayanan V. (2020). Ophthalmic diagnosis using deep learning with fundus images–A critical review. Artif. Int. Med. 102, 101758. 10.1016/j.artmed.2019.101758 31980096

[B41] Serra J. (1986). “Introduction to mathematical morphology,” in Encyclopedia of Mathematical Geosciences, eds Daya Sagar B. S. Cheng Q. McKinley J. Agterberg F. (Cham: Springer International Publishing), 1–16.

[B42] Serra J. (2020). “Mathematical morphology,” in Encyclopedia of Mathematical Geosciences, eds Sagar B. S. D. Cheng Q. McKinley J. Agterberg F. (Cham: Springer International Publishing), 1–16.

[B43] Serra J. Soille P. (2012). Mathematical Morphology and Its Applications to Image Processing, Vol. 2. Cham: Springer Science and Business Media.

[B44] Shorten C. Khoshgoftaar T. M. (2019). A survey on image data augmentation for deep learning. J. Big Data 6, 1–48. 10.1186/s40537-019-0197-0 PMC8287113 34306963

[B45] Soffer S. Ben-Cohen A. Shimon O. Amitai M. M. Greenspan H. Klang E.. (2019). Convolutional neural networks for radiologic images: a radiologist's guide. Radiology 290, 590–606. 10.1148/radiol.2018180547 30694159

[B46] Souid A. Sakli N. Sakli H. (2021). Classification and predictions of lung diseases from chest x-rays using mobilenet v2. Appl. Sci. 11, 2751. 10.3390/app11062751

[B47] Sudlow C. Gallacher J. Allen N. Beral V. Burton P. Danesh J.. (2015). UK biobank: an open access resource for identifying the causes of a wide range of complex diseases of middle and old age. PLoS Med. 12, e1001779. 10.1371/journal.pmed.1001779 25826379 PMC4380465

[B48] Szegedy C. Ioffe S. Vanhoucke V. Alemi A. (2017). Inception-v4, inception-resnet and the impact of residual connections on learning. Proc. AAAI 31, 1–5. 10.1609/aaai.v31i1.11231

[B49] Szegedy C. Vanhoucke V. Ioffe S. Shlens J. Wojna Z. (2016). “Rethinking the inception architecture for computer vision,” in Proceedings of the IEEE Conference on Computer Vision and Pattern Recognition, Honolulu, HI: IEEE, 2818–2826.

[B50] Taherkhani A. Cosma G. McGinnity T. M. (2020). AdaBoost-CNN: An adaptive boosting algorithm for convolutional neural networks to classify multi-class imbalanced datasets using transfer learning. Neurocomputing 404, 351–366. 10.1016/j.neucom.2020.03.064

[B51] Van Rossum G. Drake F. L. (1995). Python Reference Manual. Amsterdam: Centrum voor Wiskunde en Informatica.

[B52] Walsh S. L. Calandriello L. Silva M. Sverzellati N. (2018). Deep learning for classifying fibrotic lung disease on high-resolution computed tomography: a case-cohort study. Lancet Resp. Med. 6, 837–845. 10.1016/S2213-2600(18)30286-8 30232049

[B53] Wang A. Wang Y. Chen Y. (2019). Hyperspectral image classification based on convolutional neural network and random forest. Remote Sensing Lett. 10, 1086–1094. 10.1080/2150704X.2019.1649736

[B54] Wang X. Peng Y. Lu L. Lu Z. Bagheri M. Summers R. M.. (2017). “Chestx-ray8: Hospital-scale chest x-ray database and benchmarks on weakly-supervised classification and localization of common thorax diseases,” in Proceedings of the IEEE Conference on Computer Vision and Pattern Recognition Honolulu, HI: IEEE, 2097–2106.

[B55] Wetzer E. Lindblad J. Sintorn I. M. Hultenby K. Sladoje N. (2018). “Towards automated multiscale imaging and analysis in TEM: Glomerulus detection by fusion of CNN and LBP maps,” in Proceedings of the European Conference on Computer Vision (ECCV) Workshops, Cham: ECCV.

[B56] Willemink M. J. Koszek W. A. Hardell C. Wu J. Fleischmann D. Harvey H.. (2020). Preparing medical imaging data for machine learning. Radiology 295, 4–15. 10.1148/radiol.2020192224 32068507 PMC7104701

[B57] Xie Y. Richmond D. (2018). “Pre-training on grayscale imagenet improves medical image classification,” in Proceedings of the European Conference on Computer Vision (ECCV) Workshops, Cham: ECCV.

[B58] Yu X. Zeng N. Liu S. Zhang Y. D. (2019). Utilization of DenseNet201 for diagnosis of breast abnormality. Mach. Vision Appl. 30, 1135–1144. 10.1007/s00138-019-01042-8

[B59] Zhang Z. Yin F. S. Liu J. Wong W. K. Tan N. M. Lee B. H.. (2010). “Origa-light: An online retinal fundus image database for glaucoma analysis and research,” in 2010 Annual International Conference of the IEEE Engineering in Medicine and Biology, Honolulu, HI: IEEE, 3065–3068. 10.1109/IEMBS.2010.5626137 21095735

[B60] Zhao M. Wang H. Han Y. Wang X. Dai H. N. Sun X.. (2021). Seens: Nuclei segmentation in pap smear images with selective edge enhancement. Future Gen. Comput. Syst. 114, 185–194. 10.1016/j.future.2020.07.045

