# Supplementary material for: Hybrid morphological-convolutional neural networks for computer-aided diagnosis
Source: Front Artif Intell. 2023 Sep 19;6:1253183. doi: 10.3389/frai.2023.1253183 (PMC10546173; doi:10.3389/frai.2023.1253183)
Supplement: Supplementary file 1 [file Data_Sheet_1.docx]

Supplementary Material

Hybrid Morphological-Convolutional Neural Networks for Computer-Aided Diagnosis

Martha Rebeca Canales-Fiscal*, José Gerardo Tamez-Peña

*** Correspondence:** Martha Rebeca Canales-Fiscal: marthar.canales@tec.mx

# Methodology

## Architecture


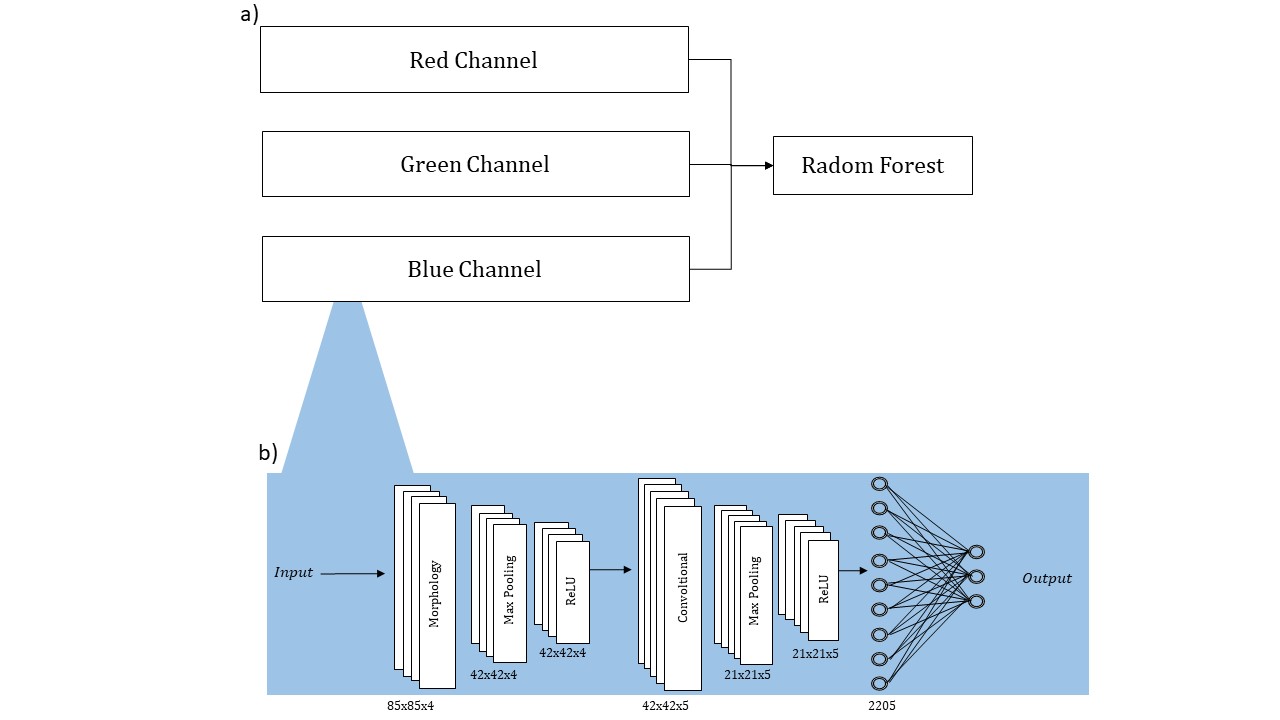


**Supplementary Figure 1.** Architecture for traffic signs classification: a) The probability outputs (a neural network per channel) are the inputs of a Random Forest classifier; b) Detailed architecture of each neural network.

## Datasets

The German Traffic Sign Recognition Benchmark (GTSRB) is a multi-class classification problem (43 classes), however, considering that we want to work with an easy classification to evaluate the general performance of our method, we limited it to two classes (1 and 13). The images come in a huge variety of sizes, for avoiding drastic image distortion, we chose images with a width of fewer than 150 pixels and resized them to 85 pixels. (Supplementary Figure 2a and 2b).


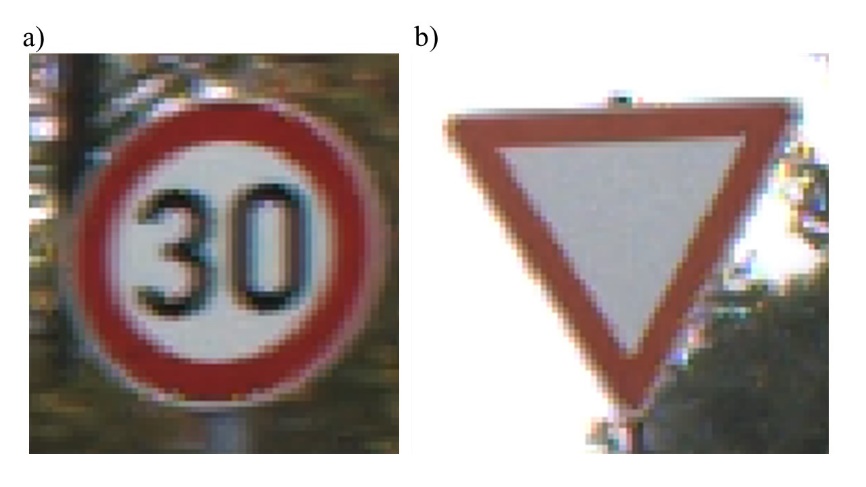


**Supplementary Figure 2.** GTSRB datasets, 2 classes chosen: a) Class 1; b) Class 13.

## Classification specifications

**Supplementary Table 1**. Parameter specification per channel-neural network per dataset

| Neural network | Regularization parameter (C) | |
| --- | --- | --- |
| ORIGA | | |
| Red NN  Green NN  Blue NN | | 1  1e^-3^  2 |
| ISIC | | |
| Red NN  Green NN  Blue NN | | 1e^-2^  1e^-2^  1e^-2^ |
| GTSRB | | |
| Red NN  Green NN  Blue NN | | 1e^-3^  1e^-3^  1e^-3^ |

# Results

## Internal evaluation


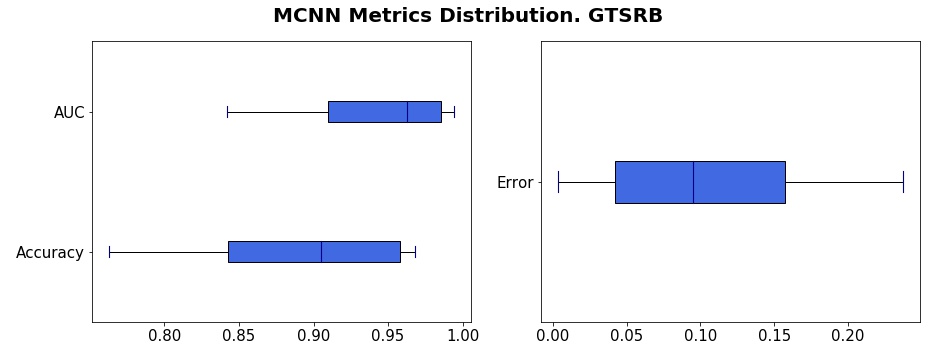


**Supplementary Figure 3.** Classification metrics distribution through 10 seeds with GTSRB dataset.

**Supplementary Table 2.** Statistical results from the 10 runs per dataset.

| Statistical metric | Accuracy | Balanced Accuracy | AUC | Error |
| --- | --- | --- | --- | --- |
| GTSRB | | | | |
| Mean  Variance  Std  Median | 0.8899  0.0058  0.0766  0.9050 | 0.8899  0.0058  0.0766  0.9050 | 0.9423  0.0030  0.0551  0.9625 | 0.1068  0.0065  0.0806  0.095 |


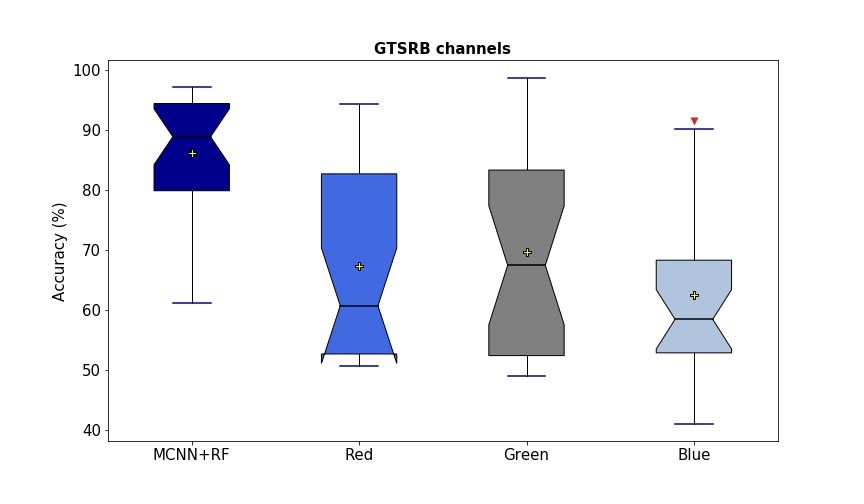


**Supplementary Figure 4.** Accuracy distributions of GTSRB classification. Complete architecture (MCNN + Random Forest) vs neural networks working with each channel (Red, Green, and Blue).

## CNN’s performance


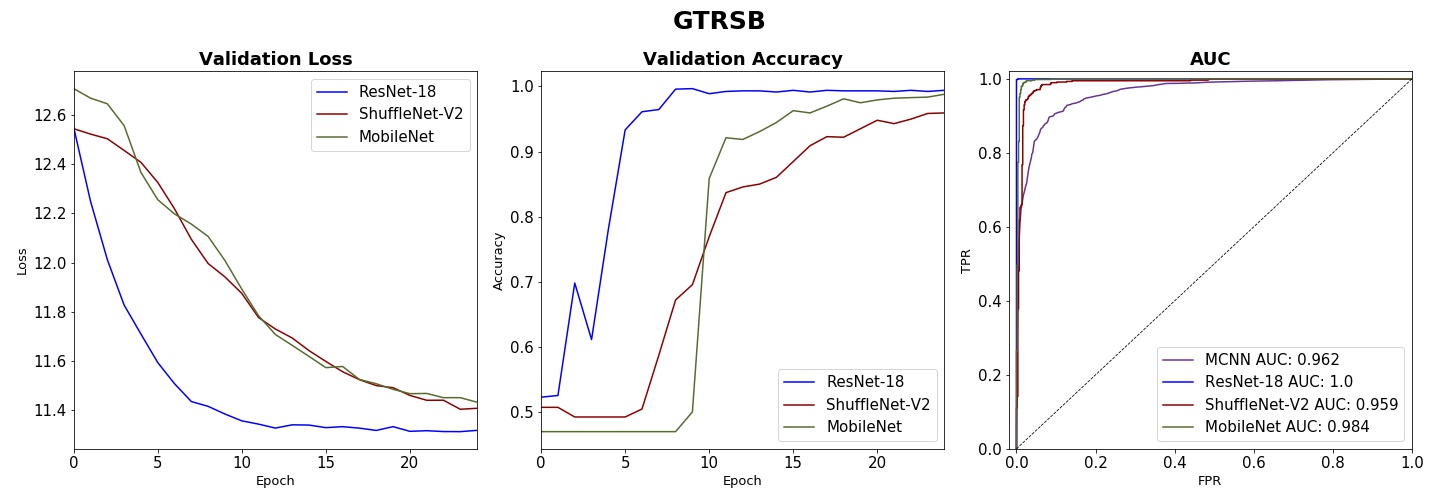


**Supplementary Figure 5.** Results for traffic signs classification using three CNNs and MCNN.
